# Supplementary material for: Lower Respiratory Tract Microbiome Signatures of Health and Lung Cancer Across Different Smoking Statuses
Source: Cancers (Basel). 2025 Aug 13;17(16):2643. doi: 10.3390/cancers17162643 (PMC12384783; doi:10.3390/cancers17162643)
Supplement: Supplementary file 1 [file cancers-17-02643-s001.zip › Supplementary Table S1.pdf]

Supplementary Table S1. Mean percentages of bacterial genera in the sputum of healthy individuals with different smoking statuses. Mann-Whitney U test with False Discovery Rate (FDR) correction.

Genus	Smokers	Former smokers	Nonsmokers	p-value		
				Smokers vs. former smokers	Smokers vs. nonsmokers	Former smokers vs. nonsmokers
Streptococcus	22.36↑	17.46	16.55	>0.05	0.01*	>0.05
Prevotella (*f. Prevotellfceae*)	16.08	16.14	15.68	>0.05	>0.05	>0.05
Veillonella	13.88	18.37	13.12	>0.05	>0.05	>0.05
Anaerosinus	11.31	15.79	8.64	>0.05	>0.05	>0.05
Actinomyces	5.2	5.09	4.81	>0.05	>0.05	>0.05
Porphyromonas	3.79	2.73	5.03	>0.05	>0.05	>0.05
Megasphaera	2.69	1.64	2.07	>0.05	>0.05	>0.05
Selenomonas	3.63	2.7	3.92	>0.05	>0.05	>0.05
Streptobacillus	2.36	1.45	2.25	>0.05	>0.05	>0.05
Leptotrichia	2.23	2.24	3.53	>0.05	>0.05	>0.05
Alloprevotella	1.94 ↓	2.41	2.76	>0.05	**0.01***	>0.05
Mycoplasma	1.93	0.96	2.84	>0.05	>0.05	>0.05
Atopobium	1.78 ↑	0.8	1.11	**0.01***	0.03	>0.05
Fusobacterium	1.78 ↓	2.35	3.92	>0.05	**0.004***	>0.05
Gemella	1.73	2.12	1.55	>0.05	>0.05	>0.05
Bacillus	1.61	1.75	1.13	>0.05	>0.05	>0.05
Rothia	1.5	2.2	1.99	>0.05	>0.05	>0.05
Granulicatella	1.21	1.12	0.58	>0.05	>0.05	>0.05
Prevotella	1.13	1.5	2.03	>0.05	>0.05	>0.05

(f. Paraprevotellacacea)						
Neisseria	1.04↓	5.39	4.49	**0.0001***	**0.00001***	>0.05
Macellibacteroides	1.03	1.45	1.44	>0.05	>0.05	>0.05
Stomatobaculum	0.89	0.49	0.47	>0.05	>0.05	>0.05
Campylobacter	0.72	1.16	1.45	>0.05	>0.05	>0.05
Bacteroides	0.71	1.13	0.78	>0.05	>0.05	>0.05
Capnocytophaga	0.67 ↓	1.01	1.27	>0.05	0.03	>0.05
Lachnoanaerobaculum	0.64	0.48	0.57	>0.05	>0.05	>0.05
Treponema	0.62	0.22	0.33	>0.05	>0.05	>0.05
Oribacterium	0.62	0.47	0.69	>0.05	>0.05	>0.05
Vestibaculum	0.62	0.19	0.45	>0.05	>0.05	>0.05
Clostridium (f.Lachnospiraceae)	0.57	0.6	0.41	>0.05	>0.05	>0.05
Peptostreptococcus	0.44	0.47	0.46	>0.05	>0.05	>0.05
Bulleidea	0.43	0.2	0.29	>0.05	>0.05	>0.05
Solobacterium	0.41	0.22	0.3	>0.05	>0.05	>0.05
Actinobacillus	0.38	0.28	0.78	>0.05	>0.05	>0.05
Clostridium (f.Clostridiaceae)	0.26	0.2	0.07	>0.05	>0.05	>0.05
Filifactor	0.24	0.11	0.17	>0.05	>0.05	>0.05
Actinomyces	0.16	0.36	0.23	>0.05	>0.05	>0.05
Bergeyella	0.15	0.24	0.32	>0.05	>0.05	>0.05
Haemophilus	0.14	0.04	0.3	>0.05	0.04	**0.01***
Lactobacillus	0.5	0.02↓	0.11	0.02	>0.05	>0.05

Dialister	0.2	0.19	0.28	>0.05	>0.05	>0.05
Catonella	0.09↓	0.23	0.22	0.03	**0.002***	>0.05
Zhouea	0.01↓	0.22	0.07	0.05	**0.02***	>0.05
Moriella	0.21	0.29	0.46	>0.05	>0.05	>0.05
Bordetella	0.06	0.08	0.08	>0.05	>0.05	>0.05
Kocuria	0.04	0.05	0.02	>0.05	>0.05	>0.05
Asholeplasma	0.2	0	0.01	>0.05	>0.05	>0.05
Defluviitalea	0.06	0.001	0.01	>0.05	>0.05	>0.05
Anaerorhabdus	0.05	0.04	0.01	>0.05	>0.05	>0.05
Pediococcus	0.1	0	0.003	>0.05	>0.05	>0.05
Olsenella	0.06	0.06	0.04	>0.05	>0.05	>0.05
Cardiobacterium	0.07	0.04	0.04	0.04	**0.002***	0.04
Staphylococcus	0.01	0.02	0.02	>0.05	>0.05	>0.05
Parvimonas	0.45	0.57	0.51	0.048	>0.05	>0.05
Johnsonella	0.3	0.24	0.18	>0.05	**0.01***	>0.05
Peptococcus	0.04	0.03	0.03	>0.05	>0.05	>0.05
Corynebacterium	0.08	0.07	0.05	>0.05	>0.05	>0.05
Spirohaeta	0.02	0.004	0.01	>0.05	>0.05	>0.05
Alloscardovia	0.05	0.08	0.08	>0.05	>0.05	>0.05
Abiotrophia	0.05	0.04	0.01	>0.05	0.04	0.046
Elizabethkinga	0.006	0.007	0.004	>0.05	>0.05	>0.05
Bifidobacterium	0.06	0.1	0.09	>0.05	>0.05	>0.05

Shuttleworthia	0.03	0.03	0.02	>0.05	>0.05	>0.05
Eggerthella	0.02	0.03	0.02	>0.05	>0.05	>0.05
Clostridium (f.Peptostreptococcacaea)	0.13	0.09	0.01	>0.05	>0.05	>0.05
Scardovia	0.008	0.02	0.02	>0.05	>0.05	>0.05
Sphingobacterium	0	0	0	>0.05	>0.05	>0.05
Mobiluncus	0.02	0.04	0.003	>0.05	>0.05	>0.05
Ruminofilibacter	0.005	0	0.02	>0.05	>0.05	>0.05
Lutibacter	0.004	0.07	0.003	0.04	>0.05	0.04
Barnesiella	0.01	0	0.001	>0.05	>0.05	>0.05
Pyramidobacter	0.001	0.04	0	>0.05	>0.05	>0.05
Gardnerella	0.008	0	0.002	>0.05	>0.05	>0.05
Succinispira	0	0	0	>0.05	>0.05	>0.05
Eggerthella	0.05	0	0.07	>0.05	>0.05	>0.05
Finegoldia	0.02	0	0	>0.05	>0.05	>0.05
Rhodococcus	0	0	0	>0.05	>0.05	>0.05
Slackia	0.005	0.001	0.01	>0.05	>0.05	>0.05
Jonquetella	0	0	0	>0.05	>0.05	>0.05
Rhizobium	0.004	0	0.007	>0.05	>0.05	>0.05
Sneathia	0.02	0	0.0004	>0.05	>0.05	>0.05
Psychrobacter	0	0	0	>0.05	>0.05	>0.05
Malus	0	0	0	>0.05	>0.05	>0.05

* p-value is less than FDR-adjusted p-value. ↑ - increase compared to nonsmokers. ↓ - decrease compared to nonsmokers
