# Supplementary material for: Lower Respiratory Tract Microbiome Signatures of Health and Lung Cancer Across Different Smoking Statuses
Source: Cancers (Basel). 2025 Aug 13;17(16):2643. doi: 10.3390/cancers17162643 (PMC12384783; doi:10.3390/cancers17162643)
Supplement: Supplementary file 1 [file cancers-17-02643-s001.zip › Supplementary Table S2.pdf]

Supplementary Table S2. Mean percentage of bacterial genera in the sputum of lung cancer patients with different smoking statuses. Mann-Whitney U test with False Discovery Rate (FDR) correction.

Genus	Smokers	Former smokers	Nonsmokers	p-value		
				Smokers vs. former smokers	Smokers vs. nonsmokers	Former smokers vs. nonsmokers
Streptococcus	27.37	31.39	23.38	>0.05	>0.05	>0.05
Prevotella *(f. Prevotellfceae)*	17.37	12.59	18.61	>0.05	>0.05	>0.05
Veillonella	13.15	8.45	13.58	>0.05	>0.05	0.04
Anaerosinus	9.95	6.57	7.87	>0.05	>0.05	>0.05
Actinomyces	3.68	2.56	4.13	>0.05	>0.05	>0.05
Porphyromonas	3.48	5.19	4.68	>0.05	>0.05	>0.05
Megasphaera	2.25	1.1	1.83	>0.05	>0.05	>0.05
Selenomonas	1.82	1.44	1.18	>0.05	0.03	>0.05
Streptobacillus	0.76	1.11	0.74	>0.05	>0.05	>0.05
Leptotrichia	1.1	1.47	1.19	>0.05	>0.05	>0.05
Alloprevotella	2.81	3.19	2.8	>0.05	>0.05	>0.05
Mycoplasma	0.63	1.08	1.37	>0.05	>0.05	>0.05
Atopobium	0.71	0.56	0.62	>0.05	>0.05	>0.05
Fusobacterium	1.49↓	1.86	1.95	>0.05	0.04	>0.05
Gemella	2.29	3.31	2.33	>0.05	>0.05	>0.05
Bacillus	1.94	3.09	2.09	>0.05	>0.05	>0.05

Rothia	1.95	1.87	2.32	>0.05	>0.05	>0.05
Granulicatella	1.28	1.47	1.59	>0.05	>0.05	>0.05
Prevotella (f. *Paraprevotellacacea)*	1.87	2.38	2.26	>0.05	>0.05	>0.05
Neisseria	2.86↓	5.37	3.67	>0.05	**0.01***	>0.05
Macellibacteroides	1.66	3.58	2.77	**0.02***	>0.05	>0.05
Stomatobaculum	0.29	0.41	0.39	>0.05	>0.05	>0.05
Campylobacter	0.71	1.04	0.93	>0.05	>0.05	>0.05
Bacteroides	0.84	1.53	0.45	>0.05	>0.05	>0.05
Capnocytophaga	1.34↓	5.78	1.65	0.007	>0.05	>0.05
Lachnoanaerobaculum	0.27	0.59	0.39	**0.01***	>0.05	0.03
Treponema	0.18	0.33	0.08	>0.05	>0.05	>0.05
Oribacterium	0.62	0.39	0.44	>0.05	>0.05	>0.05
Vestibaculum	0.61	0.08	0.34	>0.05	>0.05	>0.05
Clostridium (f. Lachnospiraceae)	0.3	0.34	0.47	>0.05	>0.05	>0.05
Peptostreptococcus	0.27	0.59	0.36	>0.05	>0.05	>0.05
Bulleidea	0.3	0.45	0.36	>0.05	>0.05	>0.05
Solobacterium	0.22	0.42	0.32	>0.05	>0.05	>0.05
Actinobacillus	0.53	0.2	0.39	>0.05	>0.05	>0.05
Clostridium	0.1	0.33	0.05	0.03	>0.05	**0.02***

(f.Clostridiaceae)						
Filifactor	0.08	0.05	0.04	>0.05	>0.05	>0.05
Actinomyces	0.24	0.04	0.09	>0.05	>0.05	>0.05
Bergeyella	0.33	0.45	0.37	>0.05	>0.05	>0.05
Haemophilus	2.15	0.64	0.7	>0.05	>0.05	>0.05
Lactobacillus	0.26	0.09↓	0.52	>0.05	>0.05	>0.05
Dialister	0.2	0.01	0.19	>0.05	>0.05	>0.05
Catonella	0.03	0.08	0.07	>0.05	>0.05	>0.05
Zhouea	0.18↓	0.69	0.29	0.04	>0.05	>0.05
Moriella	0.11	0.09	0.19	>0.05	>0.05	>0.05
Bordetella	0.03	0.05	0.07	>0.05	>0.05	>0.05
Kocuria	0.04	0.34	0.01	**0.02***	>0.05	**0.001***
Asholeplasma	0.01	0.06	0	>0.05	>0.05	>0.05
Defluviitalea	0.004	0.05	0	0.005	>0.05	**0.004***
Anaerorhabdus	0.007	0.08	0.02	>0.05	>0.05	**0.004***
Pediococcus	0.05	0.003	0.01	>0.05	>0.05	>0.05
Olsenella	0.02	0.07	0.02	>0.05	>0.05	>0.05
Cardiobacterium	0.02	0.01	0.01	>0.05	>0.05	>0.05
Staphylococcus	0.25	0.001	0.01	>0.05	>0.05	>0.05
Parvimonas	0.3	0.5	0.19	>0.05	>0.05	>0.05

Johnsonella	0.04	0.14	0.02	>0.05	>0.05	>0.05
Peptococcus	0.02	0.05	0.01	>0.05	>0.05	>0.05
Corynebacterium	0.05	0.11	0.09	>0.05	>0.05	>0.05
Spirohaeta	0.01	0.04	0	>0.05	>0.05	>0.05
Alloscardovia	0.04	0	0.04	>0.05	>0.05	>0.05
Abiotrophia	0.03	0.15	0.09	**0.02***	>0.05	>0.05
Elizabethkinga	0.008	0.02	0.12	>0.05	>0.05	>0.05
Bifidobacterium	0.13	0.007	0.07	>0.05	>0.05	>0.05
Shuttleworthia	0.03	0.01	0.002	>0.05	>0.05	>0.05
Eggerthella	0.01	0	0.01	>0.05	>0.05	>0.05
Clostridium (f.Peptostreptococcacaea)	0.03	0.01	0.08	>0.05	>0.05	0.045
Scardovia	0.03	0.02	0.008	>0.05	>0.05	>0.05
Sphingobacterium	0.004	0	0	>0.05	>0.05	>0.05
Mobiluncus	0.02	0.04	0.002	>0.05	>0.05	0.04
Ruminofilibacter	0.0004	0	0	>0.05	>0.05	>0.05
Lutibacter	0.001	0	0	>0.05	>0.05	>0.05
Barnesiella	0.001	0.002	0.002	>0.05	>0.05	>0.05
Pyramidobacter	0.003	0	0	>0.05	>0.05	>0.05
Gardnerella	0.0009	0	0.009	>0.05	>0.05	>0.05
Succinispira	0.0005	0	0	>0.05	>0.05	>0.05

Eggerthella	0.02	0	0.01	>0.05	>0.05	>0.05
Finegoldia	0	0	0.001	>0.05	0.03	>0.05
Rhodococcus	0	0	0.002	>0.05	>0.05	>0.05
Slackia	0.01	0	0.004	>0.05	>0.05	>0.05
Jonquetella	0.0001	0	0	>0.05	>0.05	>0.05
Rhizobium	0	0	0	>0.05	>0.05	>0.05
Sneathia	0.008	0	0.04	>0.05	>0.05	>0.05
Psychrobacter	0	0	0.008	>0.05	>0.05	>0.05
Malus	0.004	0	0.001	>0.05	>0.05	>0.05

* p-value is less than FDR-adjusted p-value

↑ - increase compared nonsmokers

↓ - decrease compared to nonsmokers
