# Supplementary material for: Lower Respiratory Tract Microbiome Signatures of Health and Lung Cancer Across Different Smoking Statuses
Source: Cancers (Basel). 2025 Aug 13;17(16):2643. doi: 10.3390/cancers17162643 (PMC12384783; doi:10.3390/cancers17162643)
Supplement: Supplementary file 1 [file cancers-17-02643-s001.zip › Supplementary Table S3.pdf]

Supplementary Table S3. Differences in *Neisseria* abundance in smoking and nonsmoking patients with different histological subtypes of lung cancer.

Histological subtype	Smokers	Nonsmokers	p-value
Adenocarcinoma	3.89	4.84	**0.03**
Squamous cell carcinoma	2.7	4.24	**0.04**
