# Supplementary material for: Lower Respiratory Tract Microbiome Signatures of Health and Lung Cancer Across Different Smoking Statuses
Source: Cancers (Basel). 2025 Aug 13;17(16):2643. doi: 10.3390/cancers17162643 (PMC12384783; doi:10.3390/cancers17162643)
Supplement: Supplementary file 1 [file cancers-17-02643-s001.zip › Supplementary Table S4.pdf]

Supplementary Table S4. Spearman's correlation analysis between smoking intensity (pack-years) and bacterial abundance in the sputum.

Lung cancer smokers		Healthy smokers	
Filum/Genus	r	Filum/Genus	r
Firmicutes	-0.120424	Firmicutes	0.041494
Bacteroidetes	0.099149	Bacteroidetes	0.208810
Proteobacteria	-0.040639	Proteobacteria	-0.074951
Actinobacteria	0.073701	Actinobacteria	-0.162866
Fusobacteria	0.055682	Fusobacteria	0.126703
TM7	0.192774	TM7	0.014306
Spirochaetes	0.079674	Spirochaetes	0.060150
SR1	-0.001605	SR1	-0.089780
Tenericutes	-0.014360	Tenericutes	0.055107
Streptococcus	-0.115105	Streptococcus	0.086002
Alloprevotella	0.028693	Alloprevotella	-0.043196
Prevotella(f.Prevotellfceae)	0.174207	Prevotella(f.Prevotellfceae)	0.252776
Anaerosinus	-0.030601	Anaerosinus	0.040652
Bacillus	-0.031613	Bacillus	-0.147698
Rothia	0.082448	Rothia	0.088483
Actinomyces	0.044099	Actinomyces	-0.148629
Neisseria	-0.080979	Neisseria	-0.227321
Macellibacteroides	-0.007728	Macellibacteroides	-0.097325
Streptobacillus	0.014908	Streptobacillus	0.108704
Granulicatella	0.030929	Granulicatella	0.073083
Atopobium	0.027932	Atopobium	-0.095429
Selenomonas	**0.256317**	Selenomonas	-0.028147
Clostridium (f.Lachnospiraceae)	0.163293	Clostridium (f.Lachnospiraceae)	-0.220537
Campylobacter	0.082713	Campylobacter	**-0.382932**
Treponema	0.077536	Treponema	0.063016
Prevotella (f.Paraprevotellacacea)	-0.001721	Prevotella (f.Paraprevotellacacea)	-0.129891
Lachnoanaerobaculum	-0.042751	g.Lachnoanaerobaculum	**-0.342767**
Bacteroides	0.176760	g.Bacteroides	-0.147941

Bulleidea	0.049645	Bulleidea	-0.093364
Bergeyella	-0.100332	Bergeyella	0.039586
Clostridium (f.Clostridiaceae)	0.014493	Clostridium (f.Clostridiaceae)	-0.023661
Zhouea	-0.027577	g.Zhouea	0.113406
Mycoplasma	0.009885	Mycoplasma	0.246136
Porphiromonas	0.097586	Porphiromonas	0.129056
Leptotrichia	0.054953	Leptotrichia	0.026943
Peptostreptococcus	-0.016067	Peptostreptococcus	**-0.412244**
Vestibaculum	0.144493	Vestibaculum	0.057110
Catonella	0.086413	Catonella	0.038408
Filifactor	-0.083669	Filifactor	-0.206284
Mycoplasma	-0.049782	Mycoplasma	0.043419
Actinomyces	0.017302	Actinomyces	-0.090664
Actinobacillus	-0.015730	Actinobacillus	-0.085365
Moriella	-0.039204	Moriella	-0.164500
Bordetella	-0.030210	Bordetella	-0.148813
Kocuria	0.017415	Kocuria	0.013860
Lactobacillus	0.137386	Lactobacillus	0.032676
Asholeplasma	0.092682	Asholeplasma	0.071510
Defluviitalea	-0.143614	Defluviitalea	-0.171572
Pediococcus	0.038055	Pediococcus	-0.204188
Johnsonella	-0.065823	Johnsonella	-0.206718
Peptococcus	0.031139	Peptococcus	-0.080090
Corynebacterium	0.017352	Corynebacterium	-0.062772
Alloscardovia	-0.171164	Alloscardovia	-0.062945
Abiotrophia	0.080068	Abiotrophia	-0.002966
Elizabethkinga	0.113012	Elizabethkinga	-0.206767
Bifidobacterium	-0.112203	Bifidobacterium	-0.105538
Shuttleworthia	-0.030926	Shuttleworthia	-0.302508
Eggerthella	-0.023368	Eggerthella	0.054055
Clostridium(f.Peptostreptococcaca ea)	-0.012548	Clostridium(f.Peptostreptococcaca ea)	-0.147779

Scardovia	0.046747	Scardovia	0.083376
Veillonella	0.052689	Veillonella	0.075687
Haemophilus	0.042336	Haemophilus	-0.025421
Megasphaera	0.125266	Megasphaera	-0.105957
Stomatobaculum	0.098946	Stomatobaculum	-0.061565
Gemella	0.001658	Gemella	0.141181
Fusobacterium	0.031935	Fusobacterium	0.106541
Solobacterium	0.039710	Solobacterium	-0.165761
pack-years	1.000000	pack-years	1.000000
