# Supplementary figures and images for: Lower Respiratory Tract Microbiome Signatures of Health and Lung Cancer Across Different Smoking Statuses
Source: Cancers (Basel). 2025 Aug 13;17(16):2643. doi: 10.3390/cancers17162643 (PMC12384783; doi:10.3390/cancers17162643)

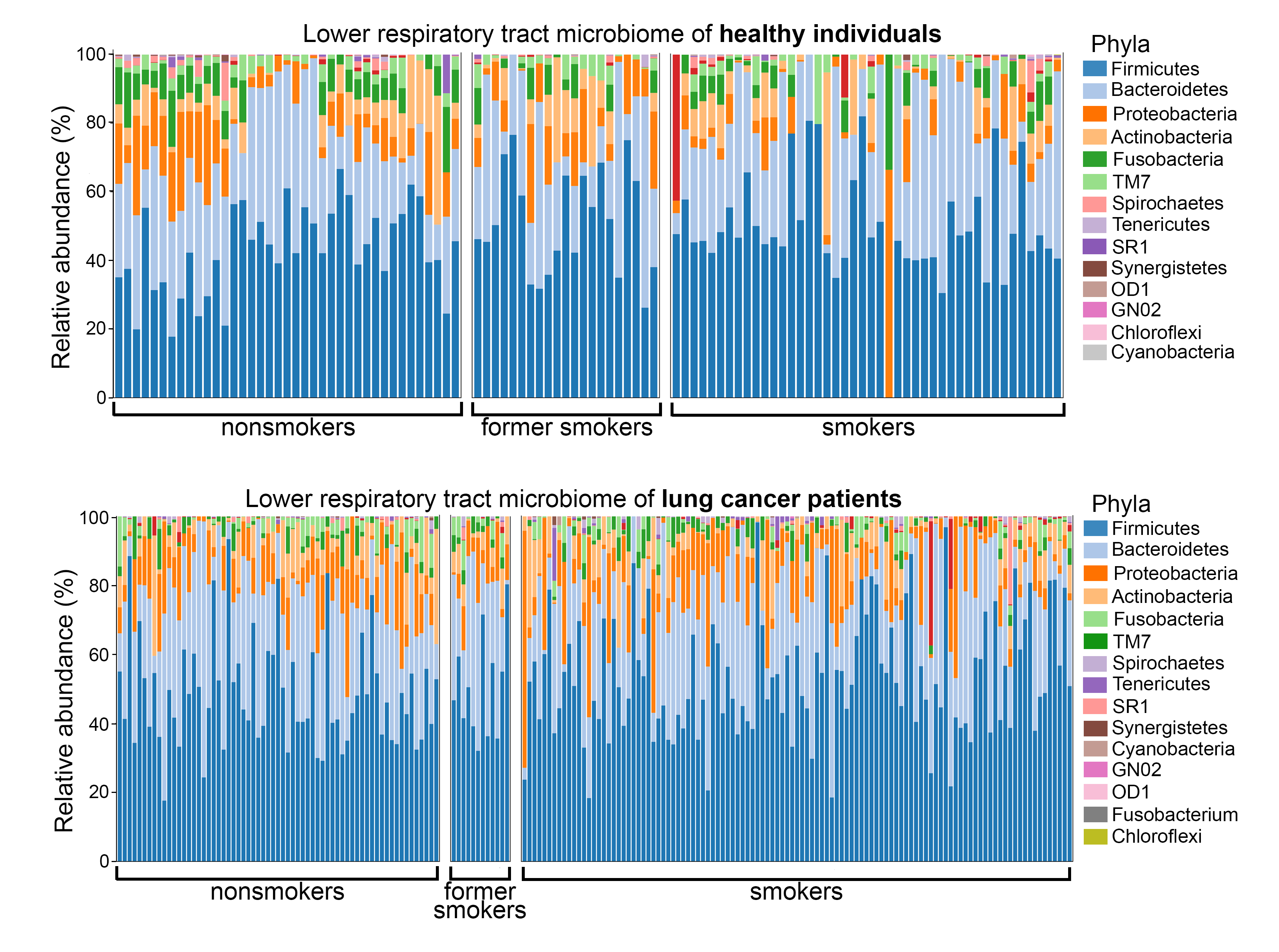

Supplement: Supplementary file 1 [file cancers-17-02643-s001.zip › Figure S1.tif]
